# Perceptions of body size, obesity threat and the willingness to lose weight among black South African adults: a qualitative study

**DOI:** 10.1186/s12889-016-3028-7

**Published:** 2016-04-29

**Authors:** Kufre Joseph Okop, Ferdinand C. Mukumbang, Thubelihle Mathole, Naomi Levitt, Thandi Puoane

**Affiliations:** School of Public Health, University of the Western Cape, Bellville, 7535 South Africa; Division of Endocrinology and Diabetes, Department of Medicine, University of Cape Town, Cape Town, South Africa

**Keywords:** Obesity, Overweight, Weight loss, Weight gain, Body image, Risk perception, Willingness, Intention, South Africa, Physical Activity

## Abstract

**Background:**

The obesity epidemic is associated with rising rates of cardiovascular disease (CVD) among adults, particularly in countries undergoing rapid urbanisation and nutrition transition. This study explored the perceptions of body size, obesity risk awareness, and the willingness to lose weight among adults in a resource-limited urban community to inform appropriate community-based interventions for the prevention of obesity.

**Method:**

This is a descriptive qualitative study. Semi-structured focus group discussions were conducted with purposively selected black men and women aged 35–70 years living in an urban South African township. Weight and height measurements were taken, and the participants were classified into optimal weight, overweight and obese groups based on their body mass index (Kg/m^2^). Participants were asked to discuss on perceived obesity threat and risk of cardiovascular disease. Information on body image perceptions and the willingness to lose excess body weight were also discussed. Discussions were conducted in the local language (isiXhosa), transcribed and translated into English. Data was analysed using the thematic analysis approach.

**Results:**

Participants generally believed that obesity could lead to health conditions such as heart attack, stroke, diabetes, and hypertension. However, severity of obesity was perceived differently in the groups. Men in all groups and women in the obese and optimal weight groups perceived obesity to be a serious threat to their health, whereas the overweight women did not. Obese participants who had experienced chronic disease conditions indicated strong perceptions of risk of obesity and cardiovascular disease. Obese participants, particularly men, expressed willingness to lose weight, compared to the men and women who were overweight. The belief that overweight is ‘normal’ and not a disease, subjective norms, and inaccessibility to physical activity facilities, negatively influenced participants’ readiness to lose weight.

**Conclusion:**

Low perception of threat of obesity to health particularly among overweight women in this community indicates a considerable challenge to obesity control. Community health education and promotion programmes that increase awareness about the risk associated with overweight, and improve the motivation for physical activity and maintenance of optimal body weight are needed.

**Electronic supplementary material:**

The online version of this article (doi:10.1186/s12889-016-3028-7) contains supplementary material, which is available to authorized users.

## Background

Obesity would affect more than 1.3 billion people globally in 2030, and is an established risk factor for cardiovascular diseases (CVD), diabetes, and all-cause mortality particularly among adults in countries undergoing rapid urbanisation and nutrition transition [1–6]. In South Africa (SA), 68 % of hypertensive disease, 45 % of ischaemic stroke, 38 % of ischaemic heart disease, and 87 % of type 2 diabetes were attributed to excess body weight (BMI > 25 kg/m^2^) in adults in 2000 [2]. As the prevalence of obesity and overweight has increased from 57 % in 2002 to 65 % in 2012 [2, 7, 8], the impact of obesity in the South African population is expected to rise considerably.

Numerous factors such as community-level, social and behavioural (mainly sedentary lifestyles combined with excess energy intake) factors are implicated in the sustained obesity epidemic in SA and other populations [9–11]. Community-level influences such as cultural perceptions of body size, the built environment, and social relationships are believed to mediate between the ‘fundamental’ or distant forces (i.e. social and economic factors, and the proximate forces (i.e. diet, physical activity and genetics) that drive obesity [10, 12]. The association between the perception of body weight or size and obesity has been explored in many populations [11, 13–15]. In SA, for example, body image perception has been associated with obesity particularly among black African women who were dissatisfied with their current body size, but perceived larger body sizes as ideal body size [16–18]. This negative body image perception has been reported to impact negatively on nutrition behaviours and weight control among black African adults [14, 15, 19]. Body image perceptions have also been reported in sub-Saharan Africa [10, 14], in the United States [13] and other populations [11, 20] to be associated with eating disorders and weight control behaviours. A study conducted in the rural communities of SA for instance, indicated that obese and overweight black women were not willing to lose weight; and very few of them had associated the food they consumed with diseases conditions such as diabetes, heart attack, stroke, cancer, or hypertension [15].

As obesity and non-communicable diseases (NCDs) burden in adults and children increases in Africa [21, 22], community-based prevention-focused interventions that seek to address the social determinants of health, particularly the socio-cultural, lifestyle and environmental factors have been recommended [12, 14, 17, 21]. Previous studies indicated that community-based interventions led to improved physical activity and intake of healthy diet, and reduction in weight among adults and school children [23–25]. Though studies looked at perceptions of body image in many African settings [10, 14, 16], information on body image perception, perceived health risk due to obesity and intention to lose weight among men and women in South African communities is limited. While Draper and her colleagues [14] explored the perceptions of body size and weight loss among adults, their focus was only on women, and the study participants were not stratified by weight categories. This study explored the perceptions of body size, obesity risk perception, and the willingness to lose weight in men and women living in a resource-limited urban community, in order to inform appropriate community-based intervention for the prevention of obesity.

### Theoretical framework

**Fig. 1:**
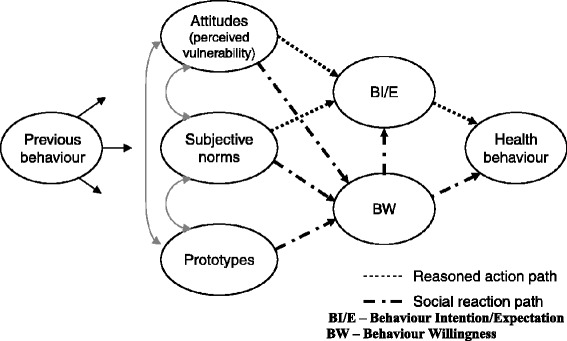
Prototype/Willingness Model (PWM). PWM suggest that previous behaviour influences attitudes, perceived vulnerability, and norms, which affect behavioural intentions and then the health behaviour

The Prototype Willingness Model (PWM) was used as the theoretical framework to guide data analysis and interpretation of this study. PWM incorporates the theory of reasoned action (TRA) and social reaction constructs (Fig. 1), and has been used to investigate the intentions to engage in health-protective and health-risk behaviours in adolescents and adults [26–28]. Empirical evidence have shown that intentions to adopt a health behaviour or treatment for health conditions (e.g. malaria, diarrhoea, and hypertension) are influenced by perceived threat or severity of such disease or consequent effect [29]. Perceived susceptibility and severity of the disease condition, and perceived benefits and barriers (including self-efficacy) substantially accounted for people’s readiness to adopt preventive health behaviours [28, 29]. In addition, TRA-based studies have shown that intentions are the primary predictors of behaviour (26, 28).

PWM posits that previous behaviour influences attitudes, perceived vulnerability, and norms that affect behavioural intentions and then the health behaviour. This model also holds that ‘prototype’, influences previous behaviour, and in turn affects willingness for health behaviour. Prototype in this model refers to ‘risk images’ or personality (i.e. significant persons) whose behaviour can be adopted by some people as ‘ideal’. The more positive people’s evaluations of the prototype and the greater their perceived similarity to the prototype, the greater will be their inclination to engage in the health-risk behaviour described in the prototype [26]. Based on PWM, the perceived threat, the reasoned actions, and the social reactions (prototype formation) together inform behaviour intentions and willingness to adopt health behaviour. This study therefore uses the PWM to investigate the influence of obesity risk perceptions on the intentions to reduce excess body weight.

## Methods

### Study design and setting

This study employed a descriptive qualitative design. Semi-structured focus group discussions (FGDs) were used to elicit views regarding perceptions about obesity risk and weight control. This study was conducted in Langa, one of the largest black communities located near Cape Town metropolis with an estimated population of 73,667 [30]. Langa is one of the resettlement communities considered to be a typical population in nutrition transition, partly because its growing population is mainly due to migration of people mostly from the rural Eastern Cape post-Apartheid. Like any other black South African townships, most residents in this community live with an average monthly household income of $200 and over 40 % are unemployed [30]. Further, 47 % of men and 60 % of women in this community had reported high school or college education [31]. Recent data showed that up to 82 % of the women and 36 % of men aged 35 to 70 years in Langa were overweight or obese based on BMI > 25.0 kg/m^2^; with only 0.6 % underweight [31].

### Sampling procedure

Purposive sampling method was used to select participants for the study from an existing Prospective Urban and Rural Epidemiology (PURE) cohort study, which examines cardiovascular risk factors and societal exposures among adults [31, 32]. The criteria for inclusion in the study were, being a female or male aged 35–70 years; and a resident of Langa community. Adults aged 35–70 years were considered for the study, because of the high burden of obesity in this age category in South African population [8]. Two research assistants approached potential participants during PURE study follow-up interviews conducted in 2014/2015 for possible participation in the study. Participants who gave verbal or signed informed consents were grouped based on sex and weight categories, and were invited to a nearby community centre on different days for discussions. Of the 89 participants who consented to participate, 78 (36 women and 42 men) returned for the group discussions. Reasons given for non-participation include having conflicting engagements at the time of the interviews. The underweight (BMI <18 Kg/m^2^) participants were excluded from the study.

### Data collection

Focus group discussions were undertaken with separate groups of women, and men based on weight category. The focus groups facilitation process is discussed in detail in the next sub-section. Prior to the group discussions, participants’ heights and weights were measured using calibrated scales and height meter with participants wearing light clothing, standing erect and without shoes. Each participant’s body mass index (BMI) was calculated in kilogramme/m squared, and their weight categories determined using standard cut-offs [33] prior to the group discussions. Participants were classified into normal (or optimal) weight (BMI 18–25 Kg/m^2^), overweight (BMI 25–30 Kg/m^2^) and obese (BMI ≥30 Kg/m^2^) groups based on their BMI. The rationale behind the separation of the groups by gender and weight status was to facilitate the comparison of views regarding obesity risk perceptions, and intention to lose weight in men and women. This could help in the provision of targeted interventions.

### Focus groups

Eight focus group discussions (FGDs) were conducted between August 2014 and February 2015. Each focus group consisted of 9–14 participants of similar sex and weight category. The number of FGDs was determined when the saturation of views was theoretically reached. The FGDs focused on body image perceptions, belief and attitude about overweight and thinness, perceived threat of overweight and awareness of obesity-related CVD risk. Semi-structured FGDs and use of body image rating figures were considered to be appropriate for the study. These methods have been proven adequate in gathering information on perceptions of body size, disease risk and weight-loss among women in this setting [14, 15, 34]. Participants’ willingness to control excess body weight was also explored.

A seven-item FGD guide (see attached Additional file 1) was used to facilitate the group discussions. After obtaining permission from the participants, the FGDs were recorded using a digital recorder, and a note-taker documented nonverbal cues and a summary of the discussions. Two indigenous graduate research assistants conducted the discussions in isiXhosa, with guidance from two of the researchers.

**Fig. 2:**
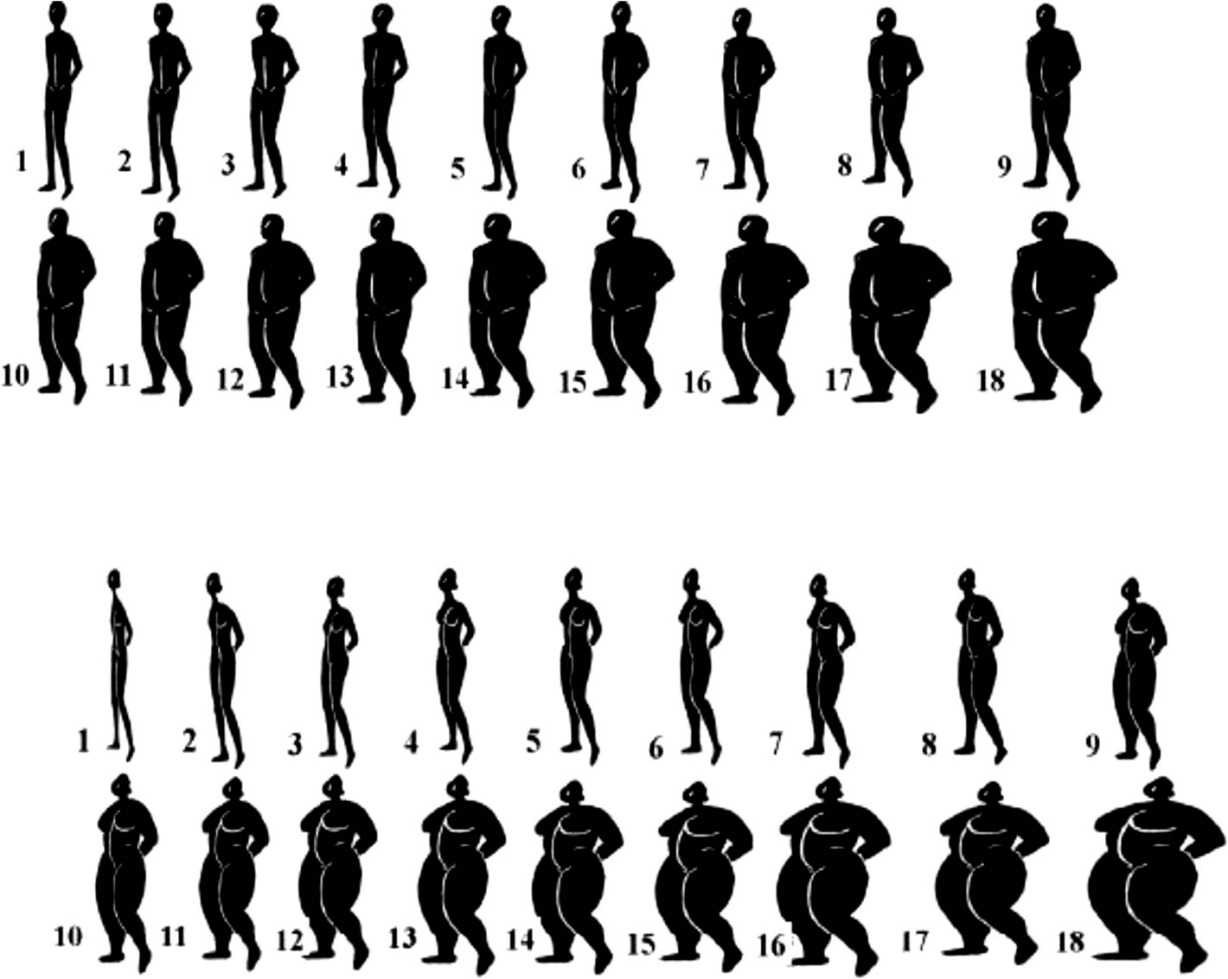
Body Image Rating Figures for men and women. Validated sex specific body image silhouettes used to assess body size perception

The discussions began with general questions on the causes of and susceptibility to obesity. To further explore the perceived threat (or severity) of obesity, participants were asked the question ‘*Do you think you may be at risk of cardiovascular disease or any health problem at your current weight?’* Since the pilot study we conducted indicated that normal weight groups were less likely to perceive any risk at their current weight, they were asked a follow-up question—‘*Do you think you may be at risk of cardiovascular disease or any health problem if you gain more weight*?’ This was to enable us compare the views in the obese and non-obese groups objectively. Following this, the sex-specific validated Body Image Rating Figures (BIRF) previously used to assess body size perception and obesity among black Africans (Fig. 2) were used to explore body size perceptions [35]. The BIRF was displayed on an A3-size paper, and each participant was asked to point to the silhouette(s) corresponding to an ideal normal body size for their gender and that of opposite gender during the discussions. Participants were also asked to identify the silhouette most closely similar to their own body sizes. The willingness to lose weight was explored through two questions: i) ‘*Would you be willing to lose weight (or maintains optimal weight)?’,* and *ii) ‘What measures have you taken to lose weight (or maintain optimal body weight)?’* Each discussion session took a maximum of 90 min. No incentives were given to the participants, but snacks and transport stipend were provided.

### Data coding and analyses

Atlas.ti software was used to facilitate the coding and the organization of themes for analysis. Data was analysed using the inductive thematic analysis approach [36]. Transcripts were first hand-coded, and an initial coding framework was developed based on the identified categories and sub-themes that emerged from the data. The PWM framework guided the data coding process. In this analysis, we coded for the participants’ attitude (i.e. perceived vulnerability and threat), their subjective norms and prototypes, and explored how these components impact on their behaviour intention and willingness. Although the coding was guided by the PWM, the themes were obtained inductively [37], as we did not use a pre-determined framework to classify themes obtained. Codes with similar themes where grouped to form sub-themes. To obtain greater abstraction, sub-themes addressing similar concepts were further grouped to form the final themes, which were *obesity risk, perception and attitude to body weight, belief/norms, major disease conditions, perceived consequences, and willingness to reduce weight*.

### Validity of the study

A number of steps were taken to ensure validity and rigour in the study. The discussions were first transcribed verbatim into isiXhosa, and translated into English. Two local trained research assistants reviewed all transcripts, comparing the transcripts with the respective audio-recorded versions to ensure accuracy in the translations. Three researchers (authors: KJO, FCM, and TM) independently reviewed the transcripts, data analysis process and the emerging themes, and agreed on the final themes and theory. This process was undertaken to reduce potential lone researcher bias and to provide additional insights into themes and interpretations [38]. In addition, the summary of the discussions was read to the participants at the end of each group discussion to verify the researchers’ understanding of their views.

#### Ethics statement

The approval for the study was obtained from the research ethics committee of the University of the Western Cape. The study was explained to participants with the aid of an information sheet written in the local isiXhosa dialect. Every participant who gave verbal consent to participate in the study also completed a consent form. All information obtained during the study is kept strictly confidential.

## Results

The characteristics of the FGD participants are presented in Table 1. A total of 78 participants were recruited; 34.6 % were obese, 24.4 % overweight and 41.0 % optimal weight men and women. Findings from the study are summarised in Table 2 and presented under the following five key themes identified from the thematic analyses process: i) perceived causes of overweight; ii) attitude towards thinness and overweight; iii) body size perceptions; iv) perceived obesity threat and CVD risk; and v) willingness to lose weight. In this paper, the participants’ views are presented and discussed based on the following groups: Optimal (or normal), Overweight and Obese weight groups. Participants’ quotes are labelled based on their corresponding groups as follows: [N-Woman] for an optimal weight woman; [OO-Woman] for overweight women, and [O-Woman] obese women. Similar codes are used for the men, example [O-Man] for an obese man.

**Table 1:** Focus group participants’ age, weight and body mass index (BMI)

Participants groups	Number of participants per group	Number of Sessions	BMI (Range)	Age range (Years)	Weight range (Kg)
Men					
Obese^a^	8	1	36.5–62.9	42 to 70	77.4–96.6
Overweight^b^	10	1	25.5–31.0	36 to 64	65.3–89.2
Optimal weight^c^	10 &14	2	18.3–25.0	35 to 63	45.6–62.3
Women					
Obese^a d^	8 & 11	2	30.8–58.9	39 to 68	89.5–160.7
Overweight^b^	9	1	25.5–30.0	48 to 70	85.5–107.8
Optimal weight^c^	8	1	18.0–24.8	35 to 60	55.8–79.0
Total	78	8			

^a^Obese (BMI >30 kg/m^2^)

^b^Overweight (BMI 25–30 kg/m^2^)

^c^Optimal weight (BMI 17–25 kg/m^2^)

^d^Obese women groups included those who were considered to be grossly obese (BMI ≥ 50 Kg/m^2^)

**Table 2:** Key themes and participants views

Themes	Obese	Overweight	Normal
Causes of overweight			
Men	• Unhealthy diet/over-eating • Physical inactivity • Socio-economics status • Genetic make-up	• Unhealthy diet, • Lack of physical exercises • Fatness is culturally desirable	• Poor eating habits (junk food) • Cultural events
Women	• Over consumption of food • Eating fatty, junk and sugary foods • Inaccessibility to vegetables and fruits • Stress • Obesity comes with age • Cultural influences	• Stress • Over consumption of food • Eating fatty, junk and sugary foods • Lack of vegetables and fruits • Genetic make-up	• Poor eating habits • Unavailability of organic food • Genetic make-up • Cultural influences
Attitudes towards thinness and overweight			
Men	• Thinness attributed to sickness or disease • Overweight is culturally acceptable • Overweight associated with happiness • Excessive body fat is not desirable	• Fatness attributed to laziness, tiredness and drowsiness • Much fat can be ‘unhealthy’ • Overweight is culturally acceptable • Overweight associated with happiness	• Being skinny makes you smart, healthy and good shape • Overweight socially acceptable • Overweight associated with happiness and respect
Women	• Overweight denotes good health, dignity, happiness and respect • Thinness indicates sickness, stress, unhappiness • Associates being thin to beauty and attractive to men	• Overweight is considered ‘normal’ weight/body size • Overweight associated with happiness; Obesity not a problem if inherited • Thin people are stigmatized	• Fatness means happiness • Too much ‘fatness’ can cause sicknesses
Body size perceptions			
Men	• Unhappy with current weight/size • Uncomfortable with gaining more weight	• Satisfy with body weight • Others desire slim body sizes	• Prefer slim body size • Others desire little increase in weight
	• Larger silhouettes size 7–14 (overweight/obese categories) chosen as ideal normal size for a woman, and smaller silhouettes size 4–9 (normal/overweight) as ideal for a man		
	• Underestimate body size	• Underestimate body size	• Accurately estimate body size
Women	• Perceive current size as ‘normal’ size • Happy with current body weight/size • Grossly obese desired reduced weight—if reported personal gains of weight loss	• Desire larger body size/weight gain • Desire no weight gain—if previously suffered chronic disease.	• Dissatisfy with current body size • Desire to be overweight
	• Women chose silhouettes size 13–15 (obese) as ideal for a woman and less than size 13 (overweight or normal) for a man • Obesity is associated with women; and ‘normal’ weight associated with men		
Susceptibility to obesity			
Men	Not applicable^a^	• Vulnerable if happy and wealthy	• Susceptible to overweight if indulge in overconsumption of food
Women	Not applicable^a^	• Vulnerable to obesity if indulge in unhealthy eating	• Believe of not being susceptible
Perceived obesity risk and threat of cardiovascular diseases			
Men	• Perceived obesity as threat to health • Obesity leads to chronic conditions—high blood pressure, stroke, diabetes, cancer, and arthritis	• Chronic non-communicable disease, physical impairment, and regular pains • Obesity can lead to heart attack, • Being skinny equated to less or no health problems	• At risk of cardiovascular diseases • Obesity is not good at old age • Being fat leads to hypertension, heart attack, too much sleep
Women	• Obesity is attributed to laziness, sluggishness, stigma, and tiredness, difficulty getting size of clothing to buy • Associate obesity to diabetes and hypertension	• Low perceptions of threat • Excessive weight could cause chronic illnesses, and inactivity	• Attributes diabetes, stroke, hypertension and heart attack to overweight
Willingness to lose weight			
Men	• Indicated intention to lose weight	• Desire for personal weight loss, or maintain current body size	• Currently undertakes job-related physical activities to maintain weight
Women	• Willing to lose weight in order to reduce health risk	• Intention to gain weight/maintain current weight	• Strong intention to gain more weight

^a^Obese and overweight participants were not asked if they are susceptible to overweight

### Perceived causes of overweight

Participants were aware of the main causes of obesity and had linked obesity with diet, lifestyle and inactivity. In all the groups, participants believed that overweight can result from unhealthy diet behaviours such as eating too much fatty and starchy food, consuming lots of red meat, oil, and fried or junk food.

*Too much fat is caused by what we eat—like junk food, especially chips and fish, fatty meat, and all those things that are fried.* [N-Man]

*We eat starch and starch—for example we eat rice and potatoes at the same time. These are the things that add fat to our bodies. The other thing is ‘too much oil’—which is not good for our health.* [N-Woman]

Many also believed that, in addition to diet-related causes, overweight/obesity is hereditary, and therefore difficult to control, as captured in the words of two women in the normal weight and obese groups:

*We have big bones… Overweight is something we inherited; all of us in the family are like this. I do also understand that what we eat also plays a vital role.* [O-Woman]

*Overweight or skinny—we were created by God to be the way we are. I will never be fat because I have taken after my father as you can see that he is not fat.* [N-Woman]

Among the women, it was a common opinion that women are required (by culture) to be overweight.

*According to our values and culture, it is important for a woman to have a large body. It makes you to be respected.* [O-Woman]

Some believed that stress, lack of exercise, socio-economic status and poor access to fresh vegetables and fruits could lead to overweight. Only a few participants mentioned other causes—such as too much spices, cow liver, and high acidic food.

### Attitudes towards thinness and overweight

There were contrasting opinions on thinness and overweight across groups (Table 2), indicating that thinness and overweight have different meanings to the participants. A common opinion was that being thin was not desirable, and overweight size is socially desirable. A thin person was viewed as unhealthy, and one who suffers disease such as HIV/AIDS, TB and cancer. Others consider a thin person as someone who is experiencing lots of physical or emotional stress, or depression, which is believed to impact negatively on his or her eating habit. Statements from the different groups describe these assertions to thinness.

*If you are skinny you are not healthy. When you are thin, people think you have HIV or TB.* [N-Man]

*Being thin is not good. When my child is losing weight, I would ask her about the weight that is dropping.* [OO-Woman]

*Sometimes it could be stress, or worry… Maybe he/she got lots of stress, and too much of stress makes him/her lose weight, and also not eating healthily.* [O-Woman]

Interestingly, a positive attitude about thinness was also expressed. An obese woman gave her opinions:

*Being thin is good because when you are too fat you are not healthy. What I would like is to ‘drop’* (i.e. reduce) *my weight… The health of a person that is thin is not the same as the one that is fat. A thin person is smart and attractive.* [O-Woman]

Although participants in most groups attributed being fat to happiness and affluence, the attitudes of women towards overweight and obesity tended to differ based on their weight category. For instance, the overweight and optimal weight women believed that being ‘fat’ or overweight is ‘normal’ and acceptable, provided one does not exceed ‘normal’ fatness.

*If a person is fat (overweight) we usually assume she is happy and has* (lot of) *money. It’s evident that he/she eats nicely, and a lot, and not having problems…*[OO-Woman]

*Being fat is fine, but do not exceed the ‘normal fatness’, because you will be affected by diseases.* [N-Woman].

Younger women (36 to 40 years of age) in the two overweight groups, however, challenged the persistent cultural tolerance of large body size in this community. A 36-year old woman stated her opinion this way:

*A woman these days for her health’s sake should not be overweight… In the olden days most men used to say that they are dignified when the woman is overweight. That is why we decided to be overweight and ate everything not knowing that we are putting our health in danger. However, I believe things are changing now…”* [OO-Woman]

The older men also supported this opinion, as pointed out by a statement made by a 68-year old man.

*‘We used to follow culture,* (and) *it was a good thing to be overweight—as it was a sign of respect or happiness’.* [N-Man]

### Body size perceptions

There were mixed perceptions about body size among the groups. The participants’ perceptions can be summed up in three perspectives. First, the majority of obese and overweight women choose the silohouttes that were smaller than the one equivalent to their weight, believing they are ‘normal’ or ‘moderately overweight’. Consequently, many of them expressed satisfaction with their body size.

*I am happy with the body I have now…It is lighter than before;* (when) *I was overweight.* [O-Woman]

*I don’t want it to be fatter than this because now that I have lost some weight, I can feel that my body is light…* [OO-Woman]

Second, both overweight and optimal weight women had desired large body sizes, perceiving it as attractive and ‘normal’, whereas their male counterparts desired comparatively smaller sizes (Table 2). The common views among the overweight women and optimal weight men illustrate this point:

*“This is not my ‘normal’ weight. I would be happy and I will look more attractive, if I can gain more weight.* [N-Woman]

*I’m happy with the body size I have now. I don’t wish to be overweight …* [N-Man]

Thirdly, obese women generally preferred ‘medium size’ weight (which is equivalent to overweight or moderately obese). On the other hand, obese men desired a reduction in their weight. The participants gave their views as follows:

*As for me, a large body size is not important. Someone who has a right body size is alright—I mean medium size and not a large size.* [O-Woman]

*“I would like my body to be ‘slimmer’ than what it is now, because I am sick and unhappy”.* [O-Man]

*I would love to gain weight because with the stress* (I have)*, it would be better for me.* [O-Woman]

In addition, the formerly grossly obese participants who had reported losing substantial amount of weight in recent months preferred their present body size, counting the gains of weight loss. The assertion is captured in the words of one of the obese woman aged 65 years:

*I don’t want to be fatter* (than this)*, because now that I have lost some weight, I can feel that my body is light and those parts that were painful are much better.* [O-Woman]

In selecting an ideal body weight, the men chose overweight and obese silhouettes sizes as ideal normal size for a woman and a normal to overweight sizes for a man aged 35 years and above. In contrast, women chose obese sizes as ideal for a woman and normal or overweight for a man.

### Perceptions of risk and threat of obesity and cardiovascular disease

There were marked differences in the perceptions of susceptibility and risk of obesity and cardiovascular disease among the groups. The opinions of the women and men in the different groups are stated in these excerpts:

*I don’t think I will ever be fat any more. I was once fat* (or obese) *then I suffered diabetes.* [N-Man]

*I will not be happy if I can gain weight because I’m diabetic.* [OO-Woman]

In addition, some women in the obese and overweight groups preferred optimal (or normal) weight, alleging that this would help them to avoid diseases. The following opinion from an obese woman points to this assertion:

*I would like my body not to be skinny and at the same time not to be overweight* (i.e. obese) *but be a ‘normal’ weight. Because when you are overweight you can easily have high blood pressure and when you are skinny you can easily be attacked by TB and other diseases.* [O-Woman]

Women, particularly those in the obese and optimal weight groups believed obesity is a threat to one’s health, whereas those in overweight groups did not perceive possible risk of obesity. The overweight women who had been sick of diabetes, hypertension or other non-communicable diseases (NCDs) had expressed perceived risk of obesity. The men in most groups perceived overweight or obesity as a threat to health, alleging that it leads to chronic disease conditions including diabetes and CVDs. Moreover, women were less concerned about the threat of obesity than men partly because of their experience with NCDs and perceived health risks of excessive body weight. The views regarding risk related to obesity were as follows:

*You will have many sicknesses when you are overweight (overweight), even those (sicknesses) that you were not suffering from—because of the large body size.* [OO-Man]

*It is people who are fatter than my size that are at risk of disease—because they can have high blood pressure or heart attack. …they are eating what they are not supposed to eat.* [OO-Woman]

Participants generally associated chronic non-communicable diseases such as heart attacks, strokes, diabetes and hypertension with obesity and not overweight. In addition, participants listed some effects of obesity to include: i) physical impairment, ii) sluggishness, iii) regular pains, iv) shortness of breath, v) too much sleep, and vi) the cost of new clothes to replace the undersized ones. Interestingly, the men and women who reported obesity-related complications or had expressed some benefits of personal weight loss, and others who had seen obese relatives sick of debilitating illnesses had also perceived considerable threat of obesity. The participants sum up this notion in the following statements:

*My relative who is very fat like me had serious health problems—hypertension, and arthritis.* [O-Woman]

*No, I don’t think I will ever be very fat again because I noticed that I became sick when I was ‘overweight’* (referring to obesity)*.* [OO-Woman]

*When you are fat* (obese)*, it is easy for you to have heart problems, hypertension, stroke and high cholesterol. I am currently sick of hypertension.* [OO-Man]

*I was once fat (obese), then I suffered diabetes. I don’t think I will ever be fat any more. You can see an ‘overweight’ person walking proudly, but* (you don’t know) *that* (her) *body is painful* (i.e. aching)*. I do know it from my experience”.* [OO-Woman]

### Willingness to lose excess body weight

In most of the obese groups, participants indicated the willingness to lose excess body weight in order to prevent diseases. However, the women in the overweight groups did not express the desire to lose weight. On the other hand, obese/overweight men and the obese women who were sick with NCDs particularly expressed strong desires for personal weight loss.

*It is good to do some exercises in the morning before you eat. I do run for 30 min or an hour in the morning. … But you need to do that every day so that you can reduce your weight.* [OO-Man]

*I would like to gain more weight… As I have mentioned before, I was weighing 63 kg before I got sick, today I can see that I weigh 49 kg. This weight is not making me happy at all. I would like my weight to be at least 60 Kg.* [OO-Woman]

*It is very important to try and lose weight; do exercise… and check what you eat. Now, I do not sleep immediately after food, I do house chores/work. I have also cut down on my fat meat intake.* [O-Woman]

*I do not want to be overweight* (referring to obese) *any more. I don’t know how I can get rid of it.* [O-Man]

Participants also reported taking some actions to lose weight. These actions, which are presented in Table 3, included reducing intake of fatty meat and starchy food, avoiding sleep immediately after meals and engaging in some exercises. Interestingly, overweight women mentioned smoking, and use of slimming medications as ways to lose weight, if they ever get very fat (obese). A young woman aged 36 years gave their opinions as follows:

**Table 3:** Participants’ weight-loss practices

	Actions taken to lose weight		
Group	Obese	Overweight	Normal
Men	• Reduce intake of starchy food, and fatty meat • Moderate exercises	• Consider moderate physical exercise, brisk walk • Reduce consumption of unhealthy food	• Involved in active work-related physical activity • Some exercises—walk to shops, bust/train stations
Women	• Mild physical exercise, street walk and work, house chores • Voiding sleep immediately after meals • Reduce food portion taken	• Walking, smoking, and use of slimming medications • Avoidance of fatty/junk food	• Stop sleeping after meal, • Vigorous exercise/work • Physical exercise
Both	• Self-weighting at home is uncommon	• Visit clinic for check-up, not in connection with weight check	• No self-weighing at home • Never visit clinic for weight check.

*Exercise at the gym is one way to reduce fat. Smoking and slimming can* (also) *help you.* [OO-Woman]

In all groups, no vigorous physical activity was reported among the participants. Self-weighing, and planned clinic visit to check weight were not common practices among the participants. In all the groups, however, participants reported assessing change in their own weight through feeling on their clothes (i.e. tight or loose). An obese woman gave her opinion on physical activity and personal weight assessment as follows:

*I also saw that I have lost weight looking from my clothing size. I used to wear size 44, but now I am wearing size 38—I have lost weight.* [O-Woman]

Some barriers to losing weight were listed by the participants in all the groups. These include lack of facilities and place for physical activity, poor perceptions of and motivation for physical activity among women, and lack of access to healthy food such as fruits and vegetables. There were also the complaints about increasing crime rate in the community. Additionally, some women believed that jogging in the street is like ‘chasing the air’ and perhaps not an approved norm for a woman in this community.

*But we don’t have enough facilities to train. There are no facilities for you to go and exercise so that you can get rid of all that fat—like the white people do.* [O-Man]

*…some women run in the street as if someone is chasing them. I don’t like to chase the air, though chasing that air, I’m told can help make you fit. [OO-Woman]*

## Discussion

### Inappropriate perceptions of risk of obesity

This study revealed that women, particularly those that were overweight did not only underestimate their body sizes, but had low perceptions of the threat of obesity, unlike the obese and overweight men. The overweight women had presumed their weight to be *‘normal’,* and partly because they had believed obesity not to be a debilitating disease condition like diabetes or stroke. The importance of appropriate perception of risk in improving intention and willingness for health behaviours has been reported in previous studies [26, 28]. Interestingly, the women and men in all the groups had linked excessive body weight with chronic diseases such as hypertension, diabetes, shortness of breath, heart attack, and stroke. Although most participants understood correctly the causes of obesity and appreciated the possible link between obesity and NCDs, this did not translate to increased risk perceptions or some decisive steps to control body weight, especially among the women. It was however, the obese and overweight women who had been sick of NCDs were the ones who expressed obesity threat to their health. The perception of overweight as less threatening to health, and the social desirability of overweight, particularly among women in this population could present a challenge to weight reduction interventions in this setting.

### Possible factors responsible for low perception of obesity risk

The poor perceptions of risk of obesity, especially among healthy obese and overweight women can be largely attributed to the lack of awareness about possible risk of obesity, and the poor perception of severity of overweight/obesity; which have been reported in previous studies conducted in black South African communities s [14, 15, 34]. Low perceptions of obesity risk could also be linked to the lack of access to appropriate health information on risk attributed to excess body weight in these communities [12, 17]. In this study, the participants had linked obesity with diet, lifestyle, inactivity, culture and personal values. Women generally believed that obesity is not a disease, as one who is overweight or obese is not ‘sick’ unlike a person who is diabetic. This misconception about obesity could explain why overweight women, for instance, did not perceive personal risk of obesity.

Although the prevalence of obesity and NCDs remain high in this population [39], most public health interventions including facility-based health education, and media messages, however, focus mainly on HIV/AIDS and TB and not on obesity and NCDs. Moreover, in this study community, loss of weight is attributed to sickness such as HIV/AIDS. Lack of appropriate health promotion activities hinders access to quality health information and impedes informed health decisions or reasoned actions. The situation in this study community is similar to that in a black community in the United States, for which weight control decisions among obese/overweight black American women were negatively affected, largely because they were not provided with objective health information on weight loss [40], and perhaps, risk of obesity.

The perception of threat of obesity and CVD risk may not only be influenced by the lack of obesity risk awareness and poor personal risk evaluation, but the persisting positive perceptions and attitudes towards large body size in this population. The desires toward weight gain, particularly among women, can be linked to the dominant subjective norms towards large body image of which earlier studies had reported in the rural and urban communities of South Africa [15, 34], communities in Kenya [35] and in Nigeria [41] among others. In addition, the socio-cultural norms, personal values and preferences, cultural desirability of overweight, and the stigma attached to thinness (or weight loss) pose as negative influences on risk perceptions among the study participants.

### Factors that influence willingness to control weight

Behaviour intentions and behaviour willingness according to PWM, account for actual health behaviour. From these study findings, participant’s current body weight, body image perception, economic, and socio-cultural factors influenced the willingness to lose weight. The inadequate risk perception and the unwillingness to lose weight among the overweight women can be explained by the fact that these women did not perceive their body size as overweight, and therefore did not indicate the need for weight loss. Inappropriate body image perceptions as seen in this study, have been shown to hinder the adoption of weight-loss intervention among women in another township located near Cape Town, as reported by Draper and colleagues [14]. In contrast, however, findings from a study conducted in Seychelles indicated that accurate estimation of body size led to appropriate weight-control behaviours among adolescents [20].

The report of the use of slimming medications and smoking among the overweight women to maintain weight, can also be a possible explanation for low perceived vulnerability and threat among the overweight women. Findings from a study conducted in a rural South African village in the Kwazulu Natal Province also indicated that not only overweight, but obese black women were unwilling to lose weight. Nevertheless, the majority of these women did not associated poor eating habits to chronic disease conditions such as diabetes, heart attack, stroke, cancer, and hypertension [15]. The similarity in the economic and socio-cultural characteristics of this study community to that of the rural Kwazulu natal study community could also explain the seemingly similar trend of unwillingness to lose weight among overweight women in both settings. From the above findings, it could be deduced that although our study community is located within a metropolitan city, urbanization and the socio-economic environment seemed not to influence the cultural norms towards body image and weight control.

Perceived threat of obesity or CVD risk was linked with the willingness to lose weight particularly among participants (i.e. obese women and overweight/obese men) who saw sick obese/overweight relatives experiencing chronic disease conditions. It therefore seems that perception of risk and willingness to lose weight in some individuals were enhanced by observation of persons who were sick of chronic diseases in the neighbourhood, and not merely by the influence of prototypes (i.e. image-consciousness or value placed on fat people), social reactions to subjective norms, and evaluation of expected behaviour outcome as predicted by PWM [28]. From this, it can be argued that although subjective norms (social reaction) influences individual’s attitude towards a particular health behaviour, personal observation of persons affected by disease and perception of severity of that disease could enhance behaviour intention, perhaps through informed reasoning [26].

### Public health implications and recommendations

Findings from this study have important public health implications. The fact that none of the participants go to see a doctor because of their obesity, but only go to see a doctor due to symptoms of conditions related to hypertension or diabetes should be considered a challenge to public health. Since overweight status in this setting is culturally desirable, and the people in this community believe obesity is not a disease, seeking health care for excessive body weight was not considered a priority. Moreover, obesity risk perception (in the obese groups) was common among those who are experiencing or have seen others sick of chronic diseases. Poor risk perception on the part of the people with excess body weight can result in poor personal risk assessment and would affect the intention to seek health care timeously. This may lead to an increase in the number of persons with undiagnosed obesity-related health problems and NCDs.

Interventions that can facilitate appropriate health risk awareness in obesity-burdened communities should be implemented to address the inadequate risk awareness and perceptions. From recent studies, implementing CVD risk assessment (including overweight awareness) and training using community health workers (CHWs) at community levels have been shown to increase awareness and prompt referral of individuals for health care in resource-limited settings in South Africa [42], and Bangladesh, Mexico and Guatemala [43]. There is, therefore, the need to include the minimal NCD care package that incorporates CVD risk assessment in the CHWs’ care package as the South African Department of Health launches the primary health care (PHC) re-engineering programmes in communities [44]. PHC re-engineering is a model for providing expanded health care to communities through community caregivers working in teams with health professionals in designated catchment areas.

Some of the participants mentioned the lack of healthy food choices such as fruits and vegetables in their local markets. The lack of fruits and vegetables will limit peoples’ choices to other food that may not be healthy—as reported in previous study in a community near Cape Town, where junk food and fatty food were consumed as alternatives [34]. In this regard, efforts should be made to encourage local food shops or grocery stores to stock healthy food items for access by the people. In addition, the need to develop collaborative initiatives with local communities and state (or provincial government) has been recommended as a strategy to address scarcity of healthy food, and to reduce food insecurity [45, 46]. Through organised community-supported gardens, community kitchens, farmers’ markets and grocery stores, healthy foods can be provided at comparably lower cost for increase access to healthy food [46].

Another issue of public health concern from the study is the poor attitudes, and lack of resources and motivation for physical activity, which could negatively influence intentions to lose or maintain optimal weight in this community. Evidence from a recent study indicated that women with high exercise motivation were three times more likely to lose more than 10 % body weight than those who were not properly motivated [47]. It is therefore essential to implement community-based physical exercise interventions that have strong motivation for moderate and less vigorous outdoor and indoor exercises.

Lastly, this study indicated that the willingness to maintain optimal body weight might not be a function of perceived threat of obesity or health risk only, but the effect of a number of factors such as attitudes, personal values, perceived built environment, access to health information and influence of strong cultural ideals. Obesity prevention interventions that incorporate appropriate community-level education, health promotion and behaviour change advocacy should, therefore, be implemented to support attitude modification and improved the willingness to control weight. Such interventions should objectively target improving positive body size perceptions, health risk awareness and risk appraisal among obese and overweight adults.

## Strengths and limitations of the study

The study used focus groups segregated by sex, stratified by weight categories, and included adults aged 35–70 years for which obesity is highly prevalent among black South Africans [8, 31]. The segregation of participants by weight groups (optimal, overweight or obese) is believed to have ensured effective discussions on the somewhat sensitive issue about weight management and risk of disease in this setting. The participants were purposively sampled from an urban informal setting in the Western Cape Province, and therefore represent a small proportion of the black population in South Africa. Also, majority (75 %) of the participants in this study were unemployed, and of low socio-economic status and education attainment. This could have affected their views and the perceptions about obesity-related health risk as well as their weight-loss intentions.

## Conclusion

The study revealed that overweight women did not perceive themselves to be at risk of obesity. The study findings also suggest that, perceptions of severity of and risk of obesity are influenced by interrelated factors most of which discourage weight loss. The low perceived threat and severity of obesity particularly among obese women in this community underscores a considerable challenge to obesity prevention and possible resistance to recommended weight loss interventions. Based on these findings, appropriate strategies to improve awareness of the health risk of overweight are critical. Community-based wellness events could be organised around internationally recognised events like obesity, diabetes and hypertension awareness campaigns. Implementation of community directed health education and physical activity programmes can motivation community members to maintain healthy body sizes. Finally, resources such as non-commercial community sports facilities for physical activities should be considered when planning and implementing obesity interventions in this setting.

### Data availability and materials

The datasets supporting the conclusions of this article are available in the repository (Figshare) https://figshare.com/s/d8fecfc178ef91035c3e. DOI:10.6084/m9.figshare.3124915

## Additional file

Additional file 1: Focus group discussion guide. (DOCX 20 kb)

## Abbreviations

CVD: cardiovascular disease
PWM: Prototype/Willingness Model
TRA: theory of reasoned action
FGD: focus group discussions
BMI: body mass index
BIRF: body image rating figures
N-Woman: optimal weight woman
OO-Woman: overweight women
O-Woman: obese women
O-Man: optimal weight woman
OO-Man: overweight man
O-Man: obese man
CHWs: community health workers
NCDs: non-communicable diseases
