# Supplementary material for: Perceptions of body size, obesity threat and the willingness to lose weight among black South African adults: a qualitative study
Source: BMC Public Health. 2016 Apr 29;16:365. doi: 10.1186/s12889-016-3028-7 (PMC4850665; doi:10.1186/s12889-016-3028-7)
Supplement: Additional file 1: — Focus group discussion guide. (DOCX 20 kb) [file 12889_2016_3028_MOESM1_ESM.docx]

**Appendix 1: FGD Guide**

| **Section A** | **Perceived susceptibility to further weight gain or overweight** |
| --- | --- |
|  | 1. In your opinion, what are the causes of obesity?   a) Is there a possibility that you can put on more weight to what you already have now? [*Only normal weight groups]* |
|  | If yes to (a), how do you know that you are becoming overweight or obese? |
|  | 1. a) What in your opinion can make you gain some weight or become overweight? |
|  | 1. How would you feel if you gain some more weight now? Would you be happy, unhappy or unconcerned? |
| **Section B** | **Perceived obesity severity (threat) and cardiovascular disease risk** |
|  | 3. a) ***To All groups:*** Do you think you may be at risk of any disease or health problems at your current weight?  *b)* ***To normal weight groups:*** Do you think you may be at risk of any disease or health problems if your gain more weight? |
|  | c) Please, mention the diseases for which you think you can be at risk of. *Probe further or look out for cardiovascular disease* |
|  | ***[Facilitator gives body image silhouettes on cardboard to each participants}***  4. Which of these picture(s) do you think may be associated with the risk of a heart disease? |
| **Section C** | **Body Image perceptions and attitude to body weight** |
|  | 5. a) Which of these figures do you think shows an ideal normal body size for a woman (or man)?   1. Why do you think that is the ideal weight? *Could you please explain more?* |
|  | 6. Which one of these figures do you think reflects your body size? |
| **Section D** | **Willingness to control weight gain or obesity** |
|  | 7. a) Would you be willing to lose weight (or maintain optimal weight)? |
|  | b) What measures have you taken to lose weight or maintain optimal body weight? |
